# 3D Visualization of Human Blood Vascular Networks Using Single-Domain Antibodies Directed against Endothelial Cell-Selective Adhesion Molecule (ESAM)

**DOI:** 10.3390/ijms23084369

**Published:** 2022-04-15

**Authors:** Nils Rouven Hansmeier, Ina Sophie Büschlen, Rose Yinghan Behncke, Sascha Ulferts, Radjesh Bisoendial, René Hägerling

**Affiliations:** 1 Research Group ‘Lymphovascular Medicine and Translational 3D-Histopathology’, Institute of Medical and Human Genetics, Charité, Universitätsmedizin Berlin, Augustenburger Platz 1, 13353 Berlin, Germany; nils.hansmeier@charite.de (N.R.H.); ina-sophie.bueschlen@charite.de (I.S.B.); rose.behncke@charite.de (R.Y.B.); sascha.ulferts@charite.de (S.U.); 2 Berlin Institute of Health at Charité-Universitätsmedizin Berlin, BIH Center for Regenerative Therapies, Augustenburger Platz 1, 13353 Berlin, Germany; 3 Research Group ‘Development and Disease’, Max Planck Institute for Molecular Genetics, Ihnestraße 63-73, 14195 Berlin, Germany; 4 Department of Rheumatology and Clinical Immunology, Maasstad Hospital, Maasstadweg 21, 3079 DZ Rotterdam, The Netherlands; bisoendialr@maasstadziekenhuis.nl; 5 Department of Immunology, Erasmus University Medical Center, Doctor Molewaterplein 40, 3015 GD Rotterdam, The Netherlands; 6 Berlin Institute of Health at Charité, Universitätsmedizin Berlin, BIH Academy, Clinician Scientist Program, Charitéplatz 1, 10117 Berlin, Germany

**Keywords:** ESAM, nanobodies, single-domain antibodies, light sheet imaging, 3D microscopy, blood vessel marker, histopathology, 3D reconstruction

## Abstract

High-quality three-dimensional (3D) microscopy allows detailed, unrestricted and non-destructive imaging of entire volumetric tissue specimens and can therefore increase the diagnostic accuracy of histopathological tissue analysis. However, commonly used IgG antibodies are oftentimes not applicable to 3D imaging, due to their relatively large size and consequently inadequate tissue penetration and penetration speed. The lack of suitable reagents for 3D histopathology can be overcome by an emerging class of single-domain antibodies, referred to as nanobodies (Nbs), which can facilitate rapid and superior 2D and 3D histological stainings. Here, we report the generation and experimental validation of Nbs directed against the human endothelial cell-selective adhesion molecule (hESAM), which enables spatial visualization of blood vascular networks in whole-mount 3D imaging. After analysis of Nb binding properties and quality, selected Nb clones were validated in 2D and 3D imaging approaches, demonstrating comparable staining qualities to commercially available hESAM antibodies in 2D, as well as rapid and complete staining of entire specimens in 3D. We propose that the presented hESAM-Nbs can serve as novel blood vessel markers in academic research and can potentially improve 3D histopathological diagnostics of entire human tissue specimens, leading to improved treatment and superior patient outcomes.

## 1. Introduction

The detailed visualization of tissue samples is the basis to understanding, detecting, and characterizing complex pathologies, and represents an indispensable step in modern disease diagnosis. Classical histopathology, which is the current gold standard routinely used in pathology laboratories, focuses on the analysis of representative two-dimensional (2D) physical sections of samples. Besides the intrinsic limitations of the physical sectioning process, such as tissue loss or artifacts including tissue distortion, the classical 2D histopathology process is severely limited in accuracy, integrity, and spatial information, as a thin sliced specimen section is not an adequate representation of an entire volumetric specimen [1]. To visualize and subsequently interpret the precise morphological and molecular aspects of tissue samples, three-dimensional (3D) histopathology is required, as the specimen can be visualized in its entirety, which ultimately results in increased diagnostic and predictive accuracy of the tissue analysis [2].

Standard immunofluorescence stainings are routinely based on mammalian IgG antibodies, which are used to detect distinguished biomarkers in tissue specimens [3]. However, structural and chemical properties of conventional antibodies, such as their relatively large size, poor tissue penetration, heterogeneous distribution, and strong background signal, oftentimes prevent an efficient staining of the entire sample [4]. As an alternative staining reagent, nanobodies (Nbs) have been established within the scientific community, as they can produce superior immunostaining results and feature distinct technological benefits concerning generation, production, and versatility [5]. In contrast to intricate multi-chained IgG antibodies, Nbs are single-domain antibodies (sdAbs) that are composed of the antigen-binding variable domain (VHH) of heavy-chain only antibodies (HCAb), a unique antibody class found in the Camelidae species [6]. With molecular weights varying around 15 kDa, they are the smallest naturally occurring antigen-binding molecules described, as of today [7]. Moreover, Nbs feature several advantageous properties for immunostainings, including high solubility [8], high resistance to chemical and proteolytic degradation [9], a long shelf-life [10], as well as faster tissue penetration rates compared to standard IgG antibodies [11,12]. Due to their high affinity [4], specificity, and small size [13], they can also reliably bind to epitopes, otherwise inaccessible, for conventional antibodies [14,15]. Furthermore, owing to its small size, the Nb genetic sequence can comfortably be integrated into conventional plasmid-based vectors and thus be expressed in high-yield bacterial production cultures in a time- and cost-efficient manner [7]. In addition to the animal-free production procedure, plasmid expression vectors also allow for versatile genetic modifications of the expression construct, e.g., by addition or replacement of small protein tags, regulatory protein domains, and fusion protein constructs [5].

Endothelial cell-selective adhesion molecule (ESAM) is a transmembrane glycoprotein expressed by endothelial cells, activated platelets, and megakaryocytes, where it facilitates cell–cell interactions at cell junctions [16,17,18] or regulates thrombus formation [19]. Due to its high expression in blood endothelial cells, ESAM serves as a distinctive blood vessel marker used for immunostainings in the context of vascular research [20,21]. However, although several commercial ESAM antibodies are commonly used in 2D immunofluorescence stainings, there is no commercial ESAM-staining reagent available for high-quality whole-mount 3D imaging.

To overcome the present limitations of 2D microscopy as well as the lack of 3D imaging tools visualizing ESAM-positive blood vascular networks, we generated and methodologically validated innovative Nbs targeting the human ESAM protein. These novel agents generate detailed 3D representations of blood vessels in volumetric human specimens, enabling spatial morphological analysis in academic research and potential applications in clinical histopathological diagnostics.

## 2. Results

### 2.1. Generation and Selection of hESAM-Specific Nanobodies

For the generation of high-affinity Nbs targeting the human ESAM protein (hESAM), two llamas (*Lama glama*) were subjected to a 42-day immunization protocol using a recombinant hESAM-Fc protein (Supplementary Figure S1A). The hESAM-Fc immunogen consisted of the extracellular domain of the hESAM protein (ECD, amino acids 1-247) fused to the Fc domain of the human IgG1 protein (hIgG1-Fc). Immunization resulted in two Nb phagemid libraries, each containing ≈10^8^ independent clones, which were used to perform three consecutive rounds of phage display against the recombinant hESAM-Fc antigen. After enrichment of antigen-specific phages, 285 single clones were randomly selected and analyzed by ELISA screening for the presence of hESAM-specific Nbs in their periplasmic extracts. By comparing binding efficiency to wells coated with the hESAM-Fc immunogen, the hIgG1-Fc domain alone, or empty control wells, the ELISA data identified 141 positive binders for hESAM. Subsequent sequence analysis displayed 33 unique Nb candidates, which represented 6 different B-cell lineages, according to their complementary determining region (CDR) 3. Based on the ELISA as well as the B-cell lineage data, we selected one representative Nb of each lineage for further analysis (Supplementary Figure S1B,C). However, as two candidates of the same B-cell lineage displayed robust ELISA screening data, both of these clones were included in our study.

**Figure 1:**
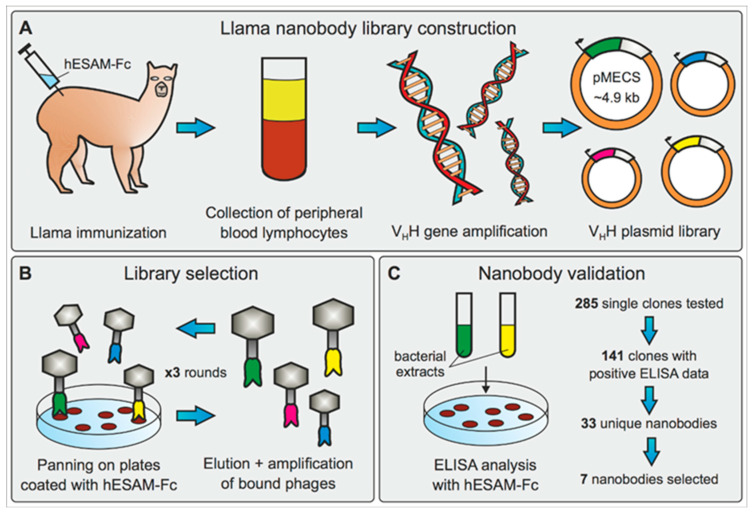
Generation and identification of hESAM-specific Nbs. (**A**) Generation of hESAM-specific Nb phagemid library by llama immunization with a recombinant hESAM-Fc antigen, followed by collection of llama peripheral blood lymphocyte total RNA and subsequent V_H_H gene amplification. Resulting V_H_H gene products were integrated into pMECS phagemid vectors to obtain a V_H_H gene plasmid library. (**B**) For selection of hESAM-specific Nbs, the phagemid library was subjected to three consecutive rounds of biopanning on plates coated with the hESAM-Fc immunogen. After each round, bound phages were eluted and amplified in *E. coli* cells. (**C**) Binding specificity of enriched candidates was assessed by ELISA screening using bacterial extracts of 285 randomly selected clones. Out of 141 positive binders, sequence analysis identified 33 unique Nbs, and 7 Nb clones were selected for further analysis.

In summary, we constructed two hESAM-specific Nb phagemid libraries, which were subjected to antigen-specific selection using phage display and ELISA screening. Out of 33 unique Nbs identified, we selected 7 Nb clones for production and assessment of 3D-histopathological imaging eligibility (Figure 1).

### 2.2. Expression and Purification of hESAM-Specific Nanobodies

**Figure 2:**
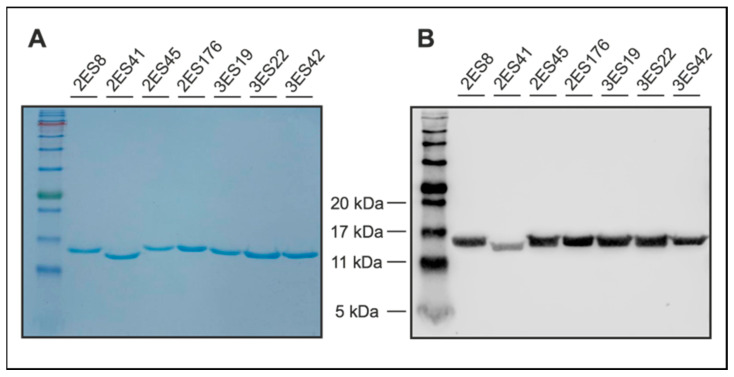
Quality control of purified hESAM-specific Nbs. (**A**) Coomassie Blue-stained SDS-PAGE of purified Nbs confirming the presence of a single protein band at standard Nb molecular weights; 5 µg of purified Nb eluates were loaded. (**B**) Western blot analysis using an anti-His IgG antibody to verify the identity of the Nbs; 5 µg of purified Nb eluates were loaded on the SDS-PAGE.

Prior to high-yield production, selected Nb sequences were recloned from the phagemid vector used for phage display (pMECS [22]) into an IPTG-inducible expression vector, which was specifically designed to produce Nbs with a C-terminal 6xHis protein tag [23]. Following overnight production in *E. coli* bacteria, Nbs were isolated in high purity, using the 6xHis protein tag in immobilized metal ion affinity chromatography (IMAC), followed by dialysis. For quality control, the purity of Nbs was evaluated by Coomassie Blue-stained SDS-PAGE, which confirmed the presence of a single protein band at the expected molecular weight for the respective Nb clones (Figure 2A). Furthermore, western blot analysis verified the identity of the Nbs by specifically detecting the 6xHis protein tag within the single protein band (Figure 2B).

Collectively, we performed high-yield production of the selected Nb clones and specific 6xHis-mediated protein purification in high purity, as demonstrated by Coomassie Blue-stained SDS-PAGE and western blot analysis. There were no unspecific bands detected by either of the methods, indicating that the Nbs were efficiently purified and thus could be tested as suitable imaging reagents.

### 2.3. Establishing 2D Nanobody Stainings in Human Skin Samples

**Figure 3:**
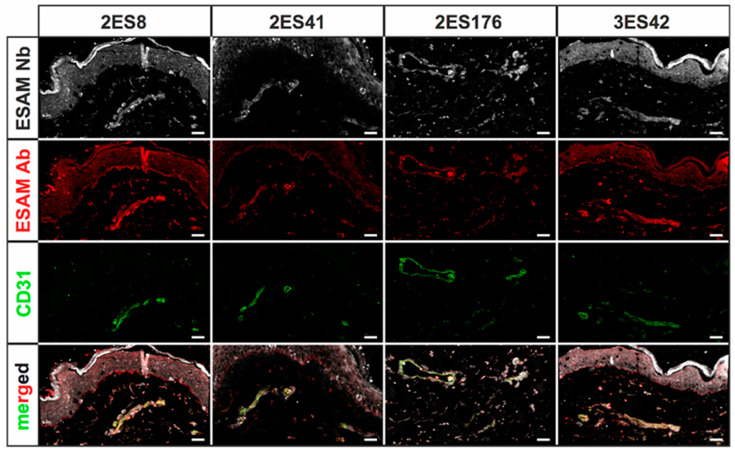
Immunofluorescence stainings of human skin cryosections using hESAM-specific Nbs. Representative images of 2D immunofluorescence stainings of 5 µm cryosections of human skin specimens stained with the respective Nbs (white), a commercially available hESAM antibody (red), and CD31 (green), an antibody visualizing blood vessels. Stained antigens are indicated next to each panel, the Nb clone used is indicated above each panel. Scale bars = 50 µm.

Before assessing whether the Nb candidates are suitable for high-quality 3D imaging approaches, the staining quality of the selected Nbs was first evaluated in standard 2D immunofluorescence stainings using 5 µm thick cryosections of human skin specimens. In order to compare staining properties, sections were not only stained with every Nb clone, but, as means of positive control, also with commercially available antibodies against hESAM and the blood vessel surface marker CD31. As expected, both control stainings visualized small intradermal blood vessels, with CD31 labeling blood endothelial cells only and the hESAM antibody additionally showing unspecific labeling of epidermal layers. Notably, stainings with the Nbs revealed specific binding to CD31-positive blood vessels for all seven clones, albeit with highly varying quality in terms of unspecific background signal. While all seven Nb clones detected distinct blood vascular structures in comparable quality to the ESAM antibody, stainings with three clones only featured weak blood vessel staining and substantial unspecific background signals, which resulted in the exclusion of these clones from further experiments (Supplementary Figure S2). However, stainings with the other four Nb clones presented visualization of blood vascular structures with similar unspecific background signals, as concomitantly detected by the hESAM antibody (Figure 3). Importantly, Nb clone ‘2ES41’ showed the most promising staining performance, as it unveiled the same structures as the ESAM antibody control, while showing significantly less unspecific background signals.

The generated Nb clones were raised against the hESAM ECD to visualize blood vascular structures in human samples; however, since the murine ESAM (mESAM) ECD showed a 67% amino acid sequence homology to hESAM ECD (Supplementary Figure S3A), we further assessed whether the Nb clones were also suitable to visualize the blood vasculature in murine specimens. To test for cross-reactivity in murine samples, we double-stained cryosections of 14.5 day-old wild type mouse embryos (E14.5) with Nb clones and a commercially available CD31 antibody. Congruent to the stainings in human skin cryosections, Nb clone ‘2ES41‘ achieved the best staining results, detecting CD31-positive endothelial cells and visualizing the morphology of blood vascular structures (Supplementary Figure S3B). Yet, other Nbs showed strong unspecific background signal and were determined to be unsuitable for high-quality microscopic imaging in mice (data not shown).

Collectively, standard 2D immunofluorescence stainings demonstrated specific visualization of blood vascular structures for all selected Nb clones in human skin cryosections. Yet, only four clones exhibited comparably low unspecific background signals, whereas three clones were excluded from further experimentations. Notably, one clone also achieved robust stainings of CD31-positive blood endothelial cells in cryosections of 14.5 day-old wild type mouse embryos.

### 2.4. Directly-Labeled Nanobodies Improve 2D Staining Quality and Incubation Time

Standard 2D immunofluorescence stainings of the selected Nbs validated their capability to specifically stain blood endothelial cell target structures and demonstrated comparable staining qualities as the commercially available hESAM control antibody. Yet, the entire staining procedure was not suitable for high-throughput imaging analysis, since the staining procedure consisted of three time-consuming staining steps: incubation with the respective Nb clone, followed by incubation with a secondary antibody binding the Nb 6xHis protein tag, and finally, incubation with a fluorescently-labeled antibody visualizing the secondary antibody. Consequently, in order to reduce incubation times, we utilized NHS-Ester chemistry to directly label the four selected Nb clones with a fluorophore, instead of using secondary and tertiary antibodies. In addition to potentially improving staining qualities, costly resources as well as multiple days of incubation periods can be evaded by avoiding secondary and tertiary staining steps.

**Figure 4:**
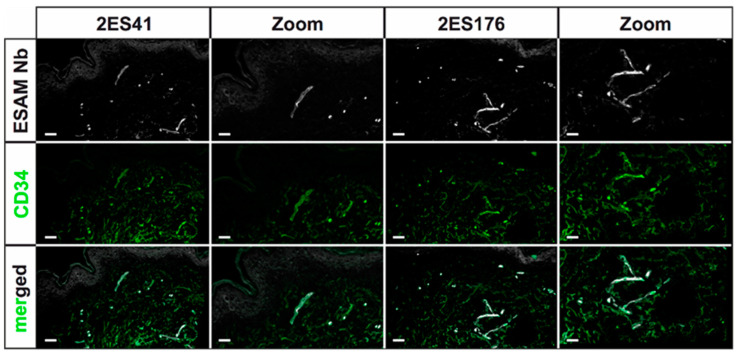
Immunofluorescence stainings of human skin cryosections using the directly-labeled hESAM-specific Nbs. Representative images of 2D immunofluorescence stainings of 5 µm cryosections of human skin specimens stained with the respective Nb (white) and CD34 (green), an antibody visualizing blood vessels. Stained antigens are indicated next to each panel, the Nb clone used is indicated above each panel. Scale bars = 50 µm.

The staining quality of directly-labeled Nbs was evaluated on 5 µm cryosections of human skin specimens, which were additionally stained with CD34, a commonly used marker for endothelial as well as hematopoetic cells [24]. As previously observed in the first 2D immunofluorescence stainings using uncoupled Nbs, the selected Nb clones, again, presented specific identification of blood vascular target structures, corroborating the preceding assessments of their eligibility for histopathological immunostainings. Of note, the strong labeling of epidermal layers as well as unspecific background signals were significantly reduced in comparison to the first standard 2D immunofluorescence stainings for two Nb clones (Figure 4). However, direct-labeling of the other two clones resulted in increased unspecific background signals, most probably due to provoked conformational changes in the Nb antigen-binding domains by fluorophore linkage (data not shown).

Taken together, the directly-labeled Nbs manifested superior staining properties, as they not only specifically visualized blood vessel target structures and produced less unspecific background signal, but also significantly reduced incubation times and were, therefore, affirmed to be suitable for rapid 3D histopathological stainings.

### 2.5. 3-Dimensional ESAM Staining of Human Skin Shows Detailed Vascular Network

Most antibodies currently used in academic and diagnostic imaging approaches are not suitable for high-quality 3D imaging technologies, since they either do not fully penetrate larger tissue samples or do not properly bind to their target structures in the harsh chemical conditions during 3D imaging sample preparation. For this reason, and owing to the fact that the commercially available hESAM antibody failed to produce high-quality 3D imaging results (data not shown), we were especially interested to validate the 3D imaging eligibility of our Nb clones in a clinically-relevant 3D imaging setting. To this end, we utilized the directly-labeled Nbs of the two selected clones in whole-mount stainings of 5 × 5 mm human skin punch biopsies and assessed if the respective Nbs were able to fully penetrate the voluminous tissue samples and specifically visualize blood vascular structures.

**Figure 5:**
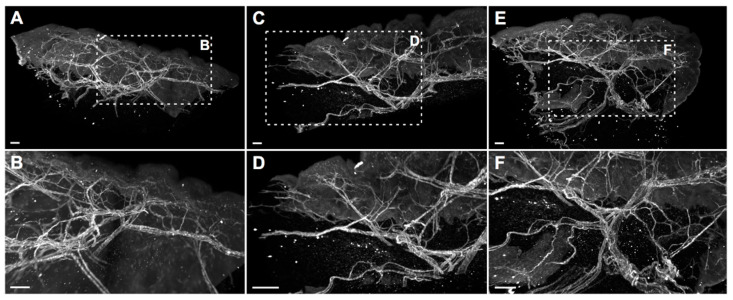
Whole-mount stainings of entire human skin biopsies using a directly-labeled hESAM-specific Nb. Whole-mount immunofluorescence stained human skin punch biopsies (5 × 5 mm) stained with the ‘2ES41’ Nb clone (white) and optically sectioned using a light sheet microscope. Shown are representative projections of 3D-rendered computer reconstructions. Overview images of three different skin biopsies are shown in the top panels (**A**,**C**,**E**), whereas the bottom panels (**B**,**D**,**F**) depict images with higher magnification of the framed areas. Scale bars = 50 µm.

Indeed, biopsy specimens stained with the directly-labeled Nbs showed precise visualization of intricate blood vascular structures within the entire tissue sample and considerably little unspecific background signal, demonstrating specific target recognition and complete Nb penetration of the voluminous samples (Supplementary Video S1). Furthermore, the delicate stainings not only allowed macroscopic representation of the entire blood vascular network, but also microscopic analysis of morphological details, such as vessel size and volume, as well as vascular branching points (Figure 5). Therefore, we confirmed that the hESAM Nb clones ‘2ES41’ and ‘2ES176’ are highly suitable for whole-mount 3D immunostainings of blood vascular networks, which can potentially improve academic and diagnostic histopathological imaging approaches.

## 3. Discussion

Thorough understanding of complex pathologies and spatial biological structures necessitates high-quality visualization of histological samples in their 3D morphological context. However, the current gold standard in histopathology still mainly relies on stainings of representative 2D physical sections due to technical limitations for 3D visualization of entire specimens, as well as the absence of suitable labeling reagents for whole-mount sample stainings. Aggravated by the usage of IgG antibodies, which are oftentimes not suitable for 3D imaging due to their relatively large size and consequently limited tissue penetration, the current 2D histopathological approach does not provide sufficient spatial information for appropriate diagnosis.

In this study, we therefore aimed to generate and experimentally validate a novel single-domain antibody (nanobody, Nb) directed against the human endothelial cell-selective adhesion molecule (hESAM), which can be used to specifically label blood endothelial cells in whole-mount 3D imaging approaches. To this end, we immunized llamas with a recombinant hESAM-Fc protein, composed of the hESAM extracellular domain (ECD) linked to the Fc domain of human IgG1, which ultimately resulted in the identification of 33 unique Nb clones. Although limited in sequence variety, as represented by only six different sequences for the complementary determining region (CDR) 3, the identified Nb clones demonstrated robust antigen-specific binding affinities in ELISA screenings. Low diversity of detected CDR3 sequences as well as the general moderate number of unique Nb clones may be caused by high glycosylation of the hESAM ECD [25,26], which might sterically hinder potential epitope recognition [27].

Human skin 2D stainings of selected Nbs reflected heterogenous results, as some Nb clones depicted strong unspecific background signals and only faint antigen-specific labeling. This finding was not unexpected, as antigen-specific ELISA screenings do not reflect the vast plethora of potential biological antigens, which may additionally be bound by the Nbs, and ultimately underlines the importance of experimental validation of every single Nb clone. Of interest, a single hESAM-Nb also exhibited moderate staining performance in 2D immunofluorescence stainings of murine embryonic tissue, highlighting the broad variety in target recognition among Nb clones with otherwise close sequence homology. Unexpectedly, however, Nb clones with sufficient staining quality in human samples also exhibited additional unspecific labeling of the epidermis, which, although also shown by the commercially available hESAM antibody control, prevents high-quality imaging. This phenomenon was bypassed through direct-labeling of selected Nbs with fluorescent dyes using NHS ester chemistry, additionally reducing incubation times and unspecific background signals. Yet, NHS ester-mediated crosslinking of fluorophores and lysine amino acids may elicit conformational changes within the inherent Nb structure, possibly resulting in altered binding affinities of specific as well as unspecific targets. Notably, we also observed changes in Nb binding specificity, since undesirable binding events were strongly enhanced in some Nb clones. Consequently, due to its unpredictable nature, NHS ester chemistry was not applicable to every Nb, so other labeling strategies may be more favorable, such as sortase- or maleimide-mediated protein labeling approaches [28,29,30]. Nonetheless, for other clones, imaging quality was considerably improved, which was demonstrated in high-quality 2D and whole-mount 3D immunofluorescence stainings. In 3D stainings of human skin biopsies, the hESAM-Nbs allowed unprecedented visualization of ESAM-positive target structures, displaying the delicate blood vascular network of the entire tissue sample.

In conclusion, in this study, we generated and experimentally validated hESAM-Nbs, which not only featured similar 2D staining properties to a commercially available IgG antibody, but also unparalleled 3D imaging quality. Previously impossible by using commercial ESAM antibodies, for the first time, the hESAM-Nbs enabled unrestricted and detailed spatial representations of ESAM-expressing blood endothelial cells in 3D imaging approaches. Owing to the animal-free, high-yield, and low-cost production, the hESAM-Nbs represent a novel and cost-efficient imaging tool for 3D histopathological analysis in academic research and potentially clinical diagnostics.

## 4. Materials and Methods

### 4.1. Llama Immunization and Construction of Nanobody Library

For the generation of Nbs targeting the hESAM protein, llamas (*Lama glama*) were immunized with the extracellular part of the hESAM protein (amino acids 1-247) fused to the Fc domain of human IgG1 (hESAM-Fc, Supplementary Figure S1A). The recombinant hESAM-Fc protein was expressed in CHO cells and purified from the CHO cell culture supernatant using Protein G Sepharose^®^ columns (ab193260, Abcam, Cambridge, UK), followed by dialysis against PBS. The immunoadjuvant used during immunizations was Adjuvant P (3111, GERBU Biotechnik GmbH, Heidelberg, Germany). In detail, two llamas were subcutaneously injected with 100 µg carrier-free, recombinant hESAM-Fc protein on days 0, 10, 16, 23, 30, and 37, followed by collection of 100 mL anti-coagulated blood on day 42 for isolation of peripheral blood lymphocytes (PBLs). For each llama, separate VHH gene libraries were constructed by using total RNA from PBLs as a template for first strand cDNA synthesis with oligo(dT) primers. Using the PBL cDNA, VHH encoding sequences were amplified via a multi-step PCR, digested with SapI (R0569, New England Biolabs, Ipswich, MA, USA), and cloned into the SapI restriction site of the pMECS phagemid vector (Prof. Serge Muyldermans, Laboratory of Cellular and Molecular Immunology, Vrije Universiteit, Brussel, Belgium). After ligation, electrocompetent *E. coli* TG1 cells (60502, Lucigen, Middleton, WI, USA) were transformed with the recombinant phagemid vectors, resulting in two VHH libraries of about 10^8^ independent transformants each. A detailed description of the workflow has been published elsewhere [22].

### 4.2. Biopanning and Screening for hESAM-Specific Nanobodies

Phage enrichment and biopanning was performed as previously described [22], with the following specifications: For the selection of hESAM-specific Nbs, three consecutive panning rounds were performed on 96-well Microtiter™ microplates (2205, Thermo Fisher Scientific, Waltham, MA, USA) coated with different concentrations of the recombinant hESAM-Fc protein (1st round: 100 µg/mL, 2nd + 3rd round: 50 µg/mL). The coating buffer was 100 mM NaHCO_3_, with pH 8.2. To reduce unspecific human IgG1-Fc binding events, the solution was supplemented with 1 µM recombinant human IgG Fc protein (110-HG, R&D Systems, Minneapolis, MN, USA). Enrichment of antigen-specific phages was about 10^2^-fold and 2 × 10^3^-fold after the 2nd and 3rd round, respectively. After each round of biopanning, *E. coli* TG1 cells were transfected with the phage output and used to amplify phages for the next round of biopanning and/or subsequent ELISA analysis. After enrichment, 285 clones were randomly selected and subjected to ELISA analysis. In detail, crude periplasmic extracts of bacterial clones were incubated on 96-well Microtiter™ microplates coated with 1 µg/mL hESAM immunogen in blocking buffer (‘hESAM’), 2 µg/mL hIgG1-Fc in blocking buffer (‘Fc’), or blocking buffer only (‘control’). The blocking buffer was 100 mM NaHCO_3_, with pH 8.2. ELISA measurements were performed as described elsewhere [22].

For expression of Nbs linked to the 6xHis protein tag only, Nb sequences were recloned from the pMECS phagemid vector into pHEN6c expression vector [23]. To this end, Nb sequences were PCR amplified using generic framework primers with the following sequences:

Forward Primer1: 5′-GATGTGCAGCTGCAGGAGTCTGGGGGAGG-3′ (all clones expect ‘3ES42’);
Forward Primer2: 5′-GATGTGCAGCTGCAGGAGTCTGGAGGAGG-3′ (‘3ES42’);
Reverse Primer: 5′-CTAGTGCGGCCGCTGAGGAGACGGTGACCTGGGT-3′ (all clones).

After purification (QIAquick PCR Purification Kit, 28104, Qiagen, Hilden, Germany), obtained PCR products were digested with PstI-HF (R3140, New England Biolabs, Ipswich, MA, USA) and BstEII-HF (R3162, New England Biolabs, Ipswich, MA, USA) restriction enzymes for 20 min at 37 °C. Simultaneously, empty pHEN6c plasmids were subjected to PstI-HF/BstEII-HF restriction digestion, additionally supplemented with 5 units of FastAP™ alkaline phosphatase (EF0651, Thermo Fisher Scientific, Waltham, MA, USA). FastAP™ activity was heat-inactivated by incubation for 5 min at 80 °C, following the incubation for 20 min at 37 °C. After purification (QIAquick PCR Purification Kit, 28104, Qiagen, Hilden, Germany), restricted Nb sequences and pHEN6c plasmids were subjected to T4 DNA igase-mediated ligation reactions at 16 °C for 16 h, containing 2.5 units of T4 DNA ligase (M0202, New England Biolabs, Ipswich, MA, USA). Subsequently, ligation products were transformed into WK6 *E. coli* cells (C303006, Thermo Fisher Scientific, Waltham, MA, USA), which were analyzed by Sanger DNA sequencing for the correct integration of the respective Nb sequences, using primers with the following sequences:

Forward Sequencing Primer: 5′-CGCCAGGGTTTTCCCAGTCACGAC-3′
Reverse Sequencing Primer: 5′-TCACACAGGAAACAGCTATGAC-3′

### 4.3. Nanobody Expression and Purification

For production of Nbs, WK6 *E. coli* harboring the pHEN6c-Nb plasmid of interest were grown in 1 L of ‘Terrific Broth’ medium (2.3 g/L KH_2_PO_4_, 16.4 g/L K_2_HPO_4_-3H_2_O, 12 g/L tryptone, 24 g/L yeast extract, 0.4% (*v/v*) glycerol) supplemented with 100 µg/mL ampicillin, 2 mM MgCl_2_, and 0.1% (*w/v*) glucose. Bacterial cultures were incubated at 37 °C with constant shaking until an OD_600_ of 0.6–0.9 was reached. Then, Nb expression was induced by adding isopropyl ß-D-1-thiogalactopyranoside (IPTG) to a final concentration of 1 mM, and cultures were incubated at 28 °C with constant shaking for 16 h. For extraction of Nbs, bacterial cells were centrifuged (8000× *g*, 8 min, RT), resuspended in 12 mL TES buffer (0.2 M Tris [pH 8.0], 0.5 mM EDTA, 0.5 M sucrose), and shaken for 1 h on ice. Subsequently, 18 mL TES/4 buffer (0.05 M Tris [pH 8.0], 0.125 mM EDTA, 0.125 M sucrose) was added to the cell solution, followed by shaking for 1 h on ice and centrifugation (8000× *g*, 30 min, 4 °C). The supernatants containing the periplasmic proteins were collected and 6xHis-tagged Nbs were purified using HIS-Select^®^ nickel affinity gel (P6611, Sigma-Aldrich, Darmstadt, Germany) according to the manufacturer’s instructions. Briefly, 2 mL HIS-Select^®^ solution was equilibrated in 48 mL of phosphate buffered saline (PBS, pH 7.4), mixed and centrifuged (2000× *g*, 2 min), followed by removal of the supernatant. The equilibration procedure was repeated for two additional times, after which bacterial periplasmic extracts were added to the equilibrated HIS-Select^®^ resin and incubated for 1 h at RT with gentle shaking. After incubation, solutions were loaded on PD-10 columns (17-0435-01, GE healthcare, Chicago, IL, USA) and washed three times with 50 mL PBS. Nbs were eluted by using 3 × 1 mL 0.5 M imidazole in PBS (I2399, Sigma-Aldrich, Darmstadt, Germany). To remove imidazole, eluted Nb solutions were subjected to overnight dialysis against PBS (3 kDa MWCO, 66382, Thermo Fisher Scientific, Waltham, MA, USA) with two changes of the dialysis buffer.

If Nb concentrations did not suffice for NHS labeling, Nb eluates were concentrated 4-fold using Pierce™ protein concentrator columns (3 kDa MWCO, 88512, Thermo Fisher Scientific, Waltham, MA, USA).

### 4.4. Coomassie-Blue Stained SDS-PAGE and Western Blotting

To evaluate sample purity, Nbs were separated via sodium dodecyl sulfate-polyacrylamide gel electrophoresis (SDS-PAGE), according to standard procedures. To this end, 5 µg of Nbs were dissolved in sample denaturation buffer (0.1 M DTT, 25 mM Tris-HCl, 14% (*w/v*) glycerol, 0.8% (*v/v*) SDS, and 0.04% (*w/v*) OrangeG in ddH_2_O) and denatured for 5 min at 96 °C. In addition to the samples, a prestained protein ladder was loaded onto the gels (ab116029, Abcam, Cambridge, UK). Following electrophoresis, proteins were either directly visualized using Coomassie staining solution (0.1% (*w/v*), Coomassie Brilliant Blue R-250 (1610400, Bio-Rad Laboratories Inc., Hercules, CA, USA), 50% (*v/v*) methanol, and 10% (*v/v*) glacial acetic acid in ddH_2_O, or blotted on a nitrocellulose membrane (1620112, Bio-Rad Laboratories Inc., Hercules, CA, USA). Coomassie Blue-stained gels were de-stained after 1 h using Coomassie destaining solution (50% ddH_2_O, 40% methanol, 10% acetic acid (*v/v/v*)). Proteins transferred on nitrocellulose membranes were detected by anti-His primary antibodies (12698, Cell Signaling Technology, Danvers, MA, USA) in combination with anti-rabbit secondary antibodies (926-32211, LI-COR Biosciences, Lincoln, NE, USA) and visualized using an Odyssey^®^ Fc Imaging System (LI-COR Biosciences, Lincoln, NE, USA).

### 4.5. Immunofluorescence Stainings

After fixation in 4% PFA in PBS, specimens were washed in PBS, embedded and snap-frozen in Tissue-Tek™ O.C.T. (12351753, Thermo Fisher Scientific, Waltham, MA, USA). Subsequently, 5 µm cryosections were fixed in ice-cold methanol for 15 min at −20 °C, 3× washed in PBS and blocked in blocking solution (5% chicken serum, 0.3% Triton™ X-100 in PBS) for 1 h at RT. Stainings were performed overnight at 4 °C using 10 µg/mL Nbs in dilution buffer (1% BSA, 1% chicken serum, 0.3% Triton™ X-100 in PBS). After 3× washing in PBS-T (0.1% (*v/v*) Tween^®^ 20 in PBS) for 10 min, the slides were incubated overnight at 4 °C using anti-His antibody (12698, Cell Signaling Technology, Danvers, MA, USA) as well as other primary antibodies for control stainings. The slides were then washed 3× in PBS-T, and finally incubated in directly-labeled anti-rabbit tertiary antibodies (A31573, Invitrogen, Waltham, MA, USA) as well as other Alexa Fluor™ dye-conjugated secondary antibodies (Invitrogen, Waltham, MA, USA) for 1 h at RT. Following 3× washing steps in PBS-T, the sections were stained in Hoechst 33342 (62249, Thermo Fisher Scientific, Waltham, MA, USA) for 1 h at RT and subsequently washed 3x in PBS-T. After samples were mounted in fluorescence mounting medium (S3023, Agilent Technologies, Santa Clara, CA, USA), representative images were captured using a LSM 780 confocal microscope (Zeiss, Oberkochen, Germany) with 20× magnification. The following antibodies were used according to the manufacturer’s instructions: rabbit monoclonal anti-His antibody (12698, Cell Signaling Technologies, Danvers, MA, USA), mouse monoclonal anti-human CD31 antibody (131M, Cell Marque Corporation, Rocklin, CA, USA), mouse monoclonal anti-human CD34 antibody (134M, Cell Marque Corporation, Rocklin, CA, USA), goat polyclonal anti-human ESAM antibody (AF2688, R&D Systems, Minneapolis, MN, USA), rat IgG2a anti-mouse PECAM-1 antibody (102502, BioLegend, San Diego, CA, USA), donkey polyclonal anti-rabbit IgG Alexa Fluor™ 647 antibody (A31573, Invitrogen, Waltham, MA, USA), donkey polyclonal anti-mouse IgG Alexa Fluor™ 488 antibody (A21202, Invitrogen, Waltham, MA, USA), donkey polyclonal anti-goat IgG Alexa Fluor™ 568 antibody (A11057, Invitrogen, Waltham, MA, USA), and donkey polyclonal anti-rat IgG Alexa Fluor™ 488 antibody (A21208, Invitrogen, Waltham, MA, USA).

### 4.6. Direct Labeling of Nanobodies

Direct coupling of hESAM Nbs with Alexa Fluor™ 647 NHS-Ester dyes (A20006, Invitrogen, Waltham, MA, USA) was performed with 1 mg of Nbs dissolved in 0.1 M NaHCO_3_ buffer (pH 8.0) and 1 mg of amine-reactive compound dissolved in DMSO. According to the manufacturer’s instructions, the reaction was incubated in the dark for 1 h at RT with continuous stirring. Finally, reaction products were isolated using a NAP™-10 gel filtration column (prepacked with Sephadex™ G-25, 17-0854-01, Thermo Fisher Scientific, Waltham, MA, USA) and eluted in PBS. For preservation purposes, sodium azide was added to the coupled Nb solution, with a final concentration of 0.03%.

### 4.7. Whole-Mount Immunofluorescence Stainings

Human skin biopsies were fixated for 4 h in 4% PFA in PBS at 4 °C, followed by permeabilization (0.5% Triton™ X-100 in PBS) at 4 °C and blocking in PermBlock solution (1% BSA, 0.5% Tween^®^ 20 in PBS) at 4 °C. Immunofluorescence stainings were performed at 4 °C with permanent shaking using directly-labeled Nbs at a final concentration of 10 µg/mL in dilution buffer (1% BSA, 1% chicken serum, 0.3% Triton™ X-100 in PBS). After 3x washing with PBS-T, stained samples were embedded in 1% low-melting point agarose and subjected to dehydration in increasing methanol concentrations (50%, 70%, 95%, >99.0%, and >99.0% (*v/v*) methanol in ddH_2_O). Finally, samples were optically cleared twice in a benzyl alcohol:benzyl benzoate solution (BABB, ratio 1:2) before being imaged on a LaVision Ultramicroscope II (LaVision BioTec, Bielefeld, Germany) at various magnifications and a step size of 2 µm. Digital 3D reconstruction of light sheet image stacks was performed using the Imaris Microscopy Image Analysis Software (Oxford Instruments, Abingdon, UK).

### 4.8. Homology Comparison of hESAM Amino Acid Sequences

For protein homology comparison, amino acid sequences of the human (ENST00000278927.10) as well as the murine (ENSMUST00000002011.14) ESAM proteins were exported from the ensemble genome browser (Ensembl release 105) and subjected to pairwise sequence alignment using the EMBOSS needle algorithm [31]. The detailed localization of the respective extracellular domains (ECDs) were obtained from the Universal Protein Resource (UniProt) database [26].

## Data Availability

Not applicable.

## Supplementary Materials

The following supporting information can be downloaded at: https://www.mdpi.com/article/10.3390/ijms23084369/s1.
