# Supplementary material for: 3D Visualization of Human Blood Vascular Networks Using Single-Domain Antibodies Directed against Endothelial Cell-Selective Adhesion Molecule (ESAM)
Source: Int J Mol Sci. 2022 Apr 15;23(8):4369. doi: 10.3390/ijms23084369 (PMC9028812; doi:10.3390/ijms23084369)
Supplement: Supplementary file 1 [file ijms-23-04369-s001.zip › Supplementary Figures.pdf]

Supplementary Figures

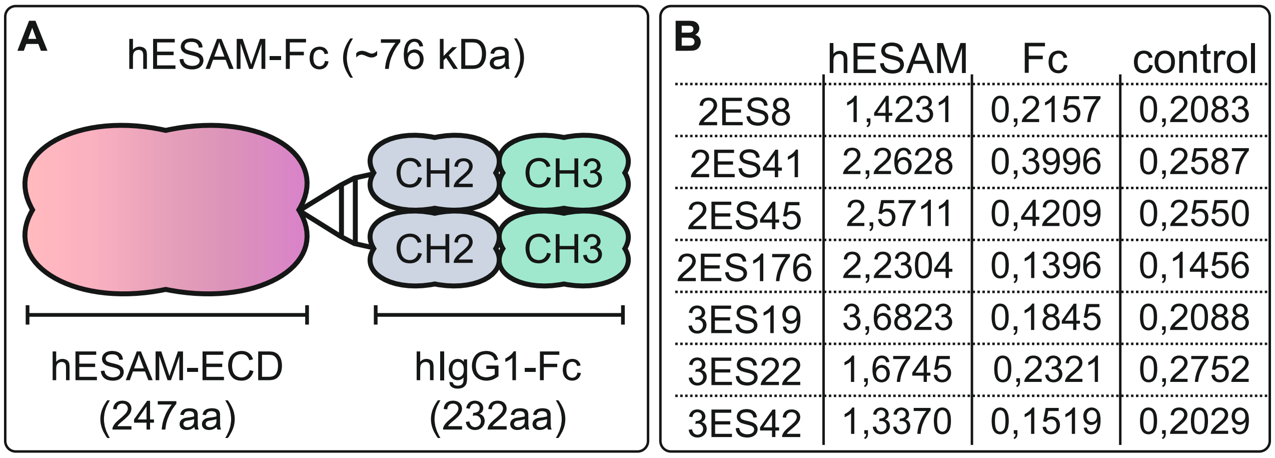

B

	hESAM	Fc	control
2ES8	1,4231	0,2157	0,2083
2ES41	2,2628	0,3996	0,2587
2ES45	2,5711	0,4209	0,2550
2ES176	2,2304	0,1396	0,1456
3ES19	3,6823	0,1845	0,2088
3ES22	1,6745	0,2321	0,2752
3ES42	1,3370	0,1519	0,2029

C

	FR3	----------CDR3-----------	FR4
2ES8	YVC	VKNK--WRTLAASSPP---EYDD	WGP
2ES41	YYC	AAAR--VNLVYAGAESAL-NYEY	WGQ
2ES45	YYC	AKNR--W-SIATAENH---EYDY	WGQ
2ES176	YYC	AATQ------GSY----RDEFEY	WGQ
3ES19	YYC	AKNR--W-SIATADNH---EYDY	WGQ
3ES22	YYC	AAKD------GSSDNIRRDDYDY	WGR
3ES42	YYC	NAHQASW-------------YDY	WGQ

Supplemental Figure S1: Characteristics of selected hESAM-specific Nbs.
*(**A**) Schematic representation of the recombinant hESAM-Fc antigen used for llama immunization. The immunogen consisted of the extracellular domain of the hESAM protein (hESAM-ECD, 247 amino acids) fused to the Fc domain of the human IgG1 protein (hIgG1-Fc, 232 amino acids).*
*(**B**) Binding efficiencies of selected Nbs assessed by whole-cell phage ELISA. Tested wells were coated with the hESAM-Fc immunogen (‚hESAM‘), the hIgG1-Fc domain alone (‚Fc‘) or only blocked (‚control‘).*
*(**C**) Amino acid sequences of the complementary determining region (CDR) 3 of selected Nb clones. [Framework region 3, FR3; Framework region 4, FR4].*

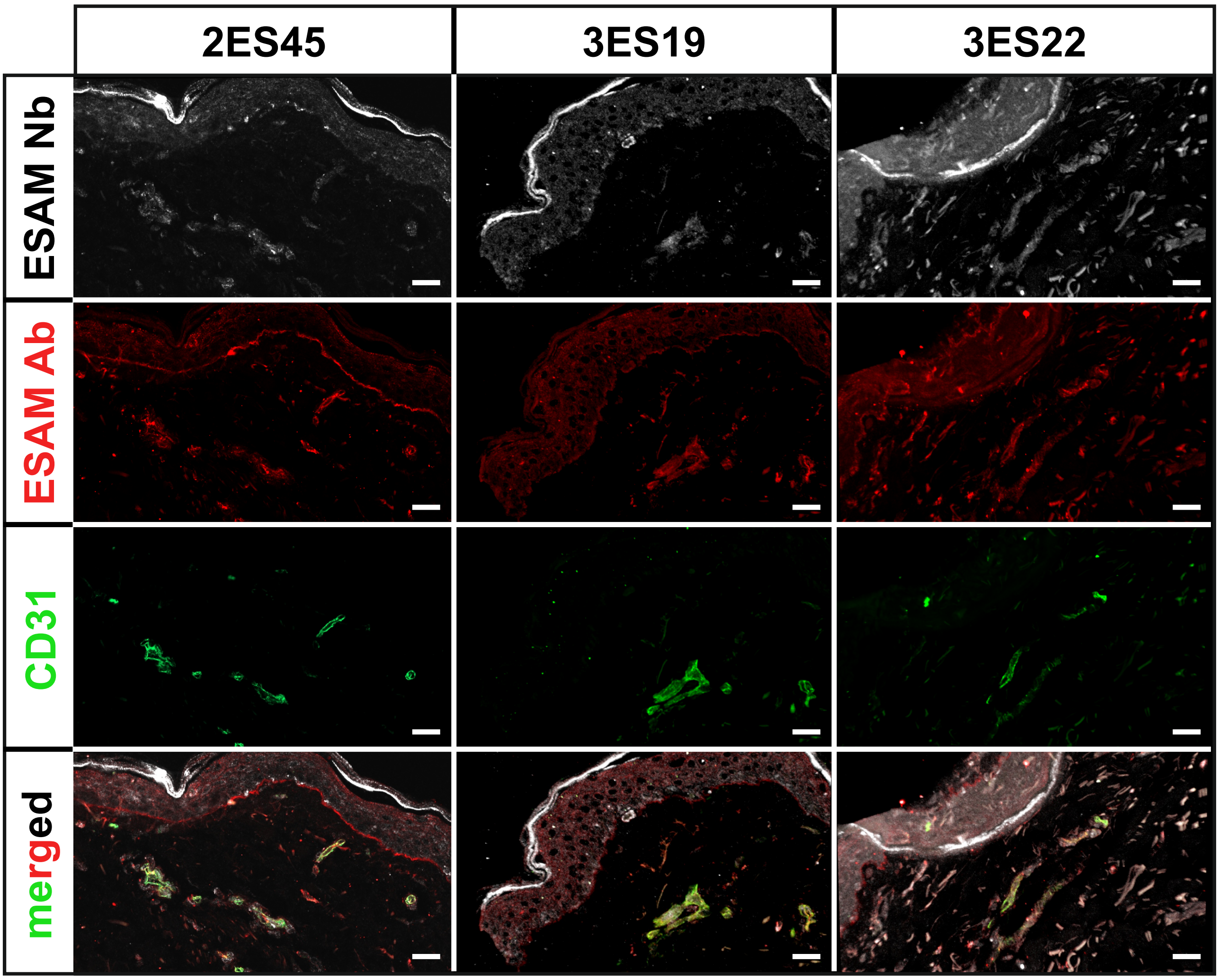

Supplementary Figure S2: Immunofluorescence stainings of human skin cryosections using the hESAM-specific Nbs ‚2ES45',3ES19' and 3ES22'.
Representative images of 2D immunofluorescence stainings of 5 µm cryosections of human skin specimens stained with the respective Nbs (white), a commercially available hESAM antibody (red) and CD31, an antibody visualizing blood vessels (green). Stained antigens are indicated next to each panel, the Nb clone is indicated above each panel. Scale bars = 50 µm.

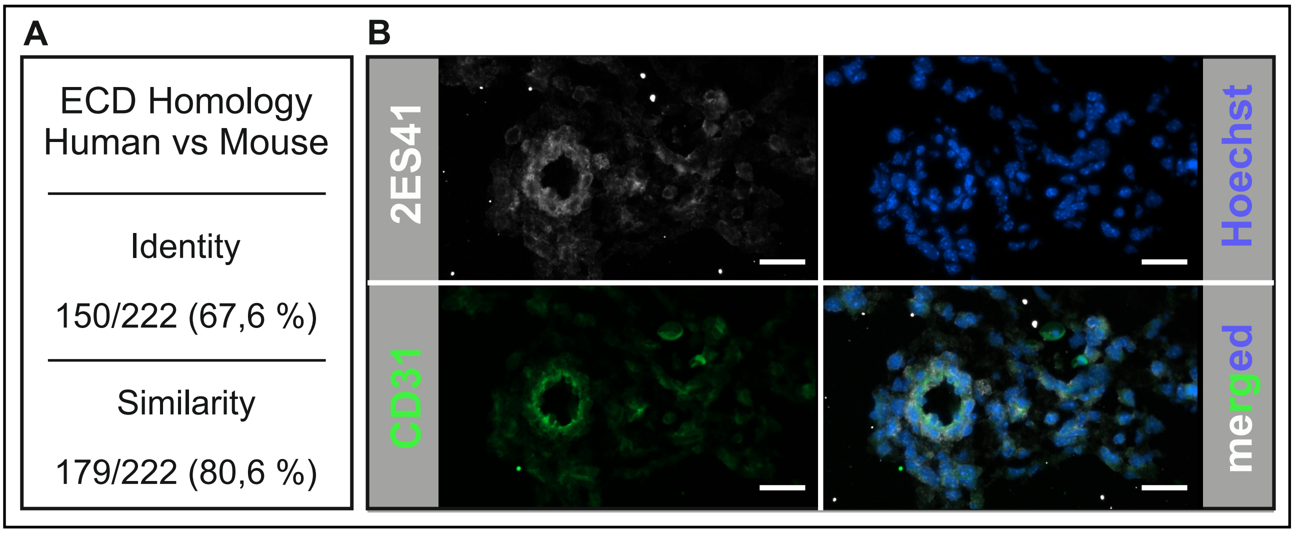

Supplemental Figure S3: Immunofluorescence staining of murine embryonic cryosections using the hESAM-specific Nb ‚2ES41‘.

*(**A**) Homology comparison of the extracellular domain (ECD) between the human and the murine ESAM amino acid sequences.*

*(**B**) Representative immunofluorescence staining of 5 µm cryosections of 14.5 day-old mouse embryos, which have been stained with the ‚2ES41‘ hESAM-Nb, blood vessel marker CD31 and nuclear staining dye ‚Hoechst‘. Stained antigens are indicated next to each panel. Scale bars = 30 µm.*

Supplemental Video: 3D reconstruction of a healthy skin biopsy revealing the blood vessel architecture using directly-labelled hESAM-specific Nbs.

A whole-mount immunostained human skin biopsy was morphologically analysed using light-sheet microscopy. Shown is a 3D reconstruction using the volume rendering software Imaris visualizing blood vessels marked by hESAM-Nb staining (white).
